# Mapping sources of chronic disease-promoting products in retail environments: An analysis of co-location patterns of alcohol, tobacco, and fast-food retailers

**DOI:** 10.1371/journal.pone.0347097

**Published:** 2026-04-20

**Authors:** Aline D’Angelo Campos, Paul L. Delamater, Marissa G. Hall, Melissa J. Cox, Shelley D. Golden

**Affiliations:** 1 Carolina Population Center, University of North Carolina at Chapel Hill, Chapel Hill, North Carolina, United States of America; 2 Department of Geography, University of North Carolina at Chapel Hill, Chapel Hill, North Carolina, United States of America; 3 Lineberger Comprehensive Cancer Center, University of North Carolina at Chapel Hill, Chapel Hill, North Carolina, United States of America; 4 Department of Health Behavior, Gillings School of Global Public Health, University of North Carolina at Chapel Hill, Chapel Hill, North Carolina, United States of America; University of Modena and Reggio Emilia: Universita degli Studi di Modena e Reggio Emilia, ITALY

## Abstract

**Objective:**

High densities of alcohol, tobacco, and fast-food retailers each pose increased risk of behaviors that contribute to chronic diseases. However, retail density studies typically examine only one type of retailer at a time. This study examines co-location patterns of alcohol, tobacco, and fast-food retailers in North Carolina, a geographically and demographically diverse US state.

**Methods:**

Using 2021 data, we calculated tract-level density of each retailer type (retailers per 1,000 residents) and categorized tracts as “high density” (≥ 75th percentile) or not in each retailer type. We then determined the percentage of residents that lived in tracts high in one, two, three, or none of the retailer types. Additionally, using bivariate logistic regression, we examined the relationship between tracts’ demographic composition and likelihood of being high in all retailer types.

**Results:**

Among individuals residing in areas with a high density of any retailer type (36.5% of the state’s population), most lived in areas simultaneously high in alcohol, tobacco, and fast-food retailers (9.7%) – exceeding those who lived in areas high in only fast food (9%), only tobacco (4.6%), or only alcohol (3.6%). Areas with a higher percentage of residents below 150% of the federal poverty line, lower median household income, or a larger African American population were more likely to be high in all three retailer types simultaneously (all p < 0.05).

**Conclusion:**

Our analysis revealed a substantial degree of co-location of alcohol, tobacco, and fast-food retailers, as well as important disparities in the demographic characteristics of the residents in areas where such retailers are more likely to co-locate. This analysis could be expanded to other states, providing insights into reinforcing retail-related risks for chronic diseases and health disparities nationwide.

## Introduction

Chronic diseases represent the leading causes of death and disability in the United States. As of 2019, cardiovascular disease, lung cancer, chronic obstructive pulmonary disease (COPD), stroke, chronic kidney disease, colorectal cancer, diabetes, and cirrhosis were among the top 10 causes of death in the country [1]. In 2022, 11.5%, 9.1%, and 6.8% of adults reported having ever been diagnosed with diabetes, heart disease, and COPD, respectively [2]. The consumption of health-harming products, especially alcoholic beverages, tobacco products, and foods with low nutritional quality, is a key modifiable risk factor for these diseases [2–4].

The retail sector is an important determinant for the consumption of alcohol, tobacco, and foods with low nutritional quality. Higher availability (i.e., prevalence) of retailers that market and sell health-harming products in an area can be an issue because it increases both exposure to (i.e., contact with) such products and their accessibility (i.e., ease of obtaining) for people in that area – which, in turn, can lead to increased consumption of such products [5,6]. Previous evidence shows that areas with higher concentrations of alcohol retailers have higher prevalence of overall alcohol consumption, drinking frequency, and adolescent alcohol use [7–9]. Similarly, higher concentrations of tobacco retailers are associated with higher prevalence of overall tobacco use, youth tobacco use, higher smoking initiation, and lower smoking cessation [10–12]. In terms of food, while exposure to health-harming foods and inaccessibility of health-promoting foods both play roles in diet quality, previous research suggests that exposure to retailers that primarily sell foods of low nutritional quality is likely the stronger predictor of diet quality and obesity in the US [13,14]. Additionally, among retailers that sell primarily unhealthy foods, fast-food restaurants are of particular interest given that they often displace higher-quality, home-cooked meals [15,16].

Most research examining the availability of alcohol, tobacco, and fast food in the US retail environment focuses exclusively on one of these products or analyzes the availability of each of these products separately. However, individuals experience retail environments as a whole, such that co-located alcohol, tobacco, and fast-food retailers expose them to and increase the accessibility of health-harming products simultaneously – thus possibly contributing to commonly co-occurring risk behaviors [17]. Previous evidence from Scotland and Germany shows that alcohol, tobacco, and fast-food retailers tend to co-locate in socially disadvantaged areas, which suggests that examining the co-location patterns of retailers offering different types of unhealthy products may help us better understand vulnerable populations’ exposure to environments that promote risk behaviors [18,19]. Yet, little is known about the degree to which alcohol, tobacco, and fast-food retailers tend to co-locate in the US. Additionally, while higher concentrations of alcohol, tobacco, and fast-food retailers are documented in areas with lower socioeconomic status and/or higher proportions of racial or ethnic minorities separately [20–22], little is known about how the co-location patterns of such retailers may relate to areas’ demographic composition in the US context.

This study examines patterns in the geographical distribution of US retailers that offer products linked to chronic disease risks. Using data from one state, we examined the degree to which alcohol, tobacco, and fast-food retailers tend to co-locate. We also examined how demographic composition relates to neighborhoods’ likelihood of having high densities of these three retailer types simultaneously.

## Methods

### Study design

This study used an ecological cross-sectional design. We used Census tracts as the unit of analysis because these contain relatively uniform population sizes while still offering a level of detail that captures variations across neighborhoods. Based on the data available, we analyzed retailer density as of January 2021. We used North Carolina as the study setting because this state’s demographic diversity, socioeconomic and racial health disparities [23], and mix of urban and rural areas provide a strong context for an analysis that can later be adapted to other states. Additionally, North Carolina’s rates of the most common chronic diseases in the US are comparable to national estimates [24]. Institutional review board approval was not required for this study as we only used publicly available ecological data.

### Data

#### Alcohol retailers.

In North Carolina, sales of alcoholic beverages are controlled by the state’s Alcohol Beverage Control (ABC) Commission. Spirits can only be sold by state-owned ABC stores and other retailers must obtain licenses to sell permitted alcoholic beverages such as beer, wine, or hard seltzer. For this study, we identified alcohol retailers using the list of businesses licensed to sell alcohol and the list of ABC stores in the state, both of which are maintained by the North Carolina ABC Commission. The list of licensed businesses was made available to us by request in October 2023, and we gathered the list of ABC stores from the North Carolina ABC Commission’s website in January 2024.

From these lists, we included retailers with at least one type of license to sell alcohol for off-premise consumption valid as of January 1^st^, 2021. We used this date to ensure temporal consistency with the latest data available for tobacco and fast-food retailers. Off-premise alcohol sales increase alcohol accessibility, being typically cheaper than on-premise sales [25] and intended for consumption at home or in private spaces, where higher quantities of alcohol per drinking occasion are common [26]. Additionally, off-premise alcohol sales conceptually parallel tobacco and fast-food sales, as all increase accessibility to health-harming products for habitual consumption.

#### Tobacco retailers.

Since the state of North Carolina does not license tobacco retailers, no list comparable to that of alcohol retailers was available. We instead followed previously established procedures to identify tobacco retailers based on store types and names [27,28]. We used a list of businesses in North Carolina as of January 2021 from the SafeGraph points-of-interest database, which included company names, addresses, geographic coordinates, and North American Industry Classification System (NAICS) codes – i.e., standard codes used by the federal government for classifying business establishments. At the time of analysis, 2021 was the most recent year for which we had available SafeGraph data.

We focused on stores that prominently market and sell tobacco near the main entrance or cashier area, making it difficult for consumers to avoid exposure to tobacco products while shopping. Based on this criterion, we initially restricted our list of tobacco retailers to “tobacco stores” (i.e., NAICS 453991), “convenience stores” (i.e., NAICS 445120), and “gasoline stations with convenience stores” (i.e., NAICS 447110). Combined, these three categories accounted for more than 65% of tobacco sales in 2017, and most establishments (i.e., more than 85%) in each of these categories sold tobacco products [29]. To avoid double-counting, we identified retailers listed under the “convenience stores” and “gasoline stations with convenience stores” categories that had overlapping addresses. We then conducted a visual Google Maps search of a random sample of 30 such retailers, which revealed each address to only contain one convenience store where we would expect tobacco to be sold. Therefore, we eliminated duplicate entries for the same address within these two categories. Lastly, we included two chain pharmacies (i.e., Walgreens and Rite Aid) and two chain dollar stores (i.e., Dollar General and Family Dollar) based on a name search, since these are prominent tobacco retailers but are classified under NAICS codes that also contain many retailers that do not sell tobacco (e.g., CVS, Dollar Tree).

We did not include supermarkets or grocery stores given that less than half of these types of retailers sell tobacco products [29]. We also did not include warehouse clubs or supercenters given that these types of retailers only account for around 10% of tobacco sales [29] and typically do not place tobacco products in high-traffic areas.

#### Fast-food retailers.

To identify fast-food retailers, we used the same list of businesses from the SafeGraph database from January 2021. We initially included retailers listed as “limited-service restaurants” (i.e., NAICS 722513), defined as “establishments primarily engaged in providing food services (…) where patrons generally order or select items and pay before eating” [30]. Upon close inspection of different categories in the Safegraph database, we found that some retailers traditionally considered as fast-food restaurants were classified as “full-service restaurants” (i.e., NAICS 722511). Given the absence of a standardized definition for fast food, we decided to include select businesses from the “full-service restaurants” category that met the following criteria: serving foods conventionally considered as meal replacements (i.e., not just snacks) and offering either only counter service or counter service with limited table service. To identify businesses that met these criteria, three independent coders went through the list of businesses in this category that had more than three locations in North Carolina in 2021 and indicated whether each of them met the criteria, did not meet the criteria, or were unknown. There were no conflicts in this coding when coders had knowledge of the business, so all businesses coded as meeting the criteria by at least one coder were included. For businesses marked as unknown by all three coders, we conducted an internet search to establish whether they met the criteria. This process led to the inclusion of 58 businesses listed as “full-service restaurants” in the SafeGraph database in our final analysis.

We did not include retailers whose primary food offerings may be of low nutritional quality but do not conventionally replace meals prepared at home (e.g., snack bars, dessert shops, bakeries, coffee shops, juice bars, convenience stores, dollar stores).

#### Demographic variables.

We obtained information on Census tracts’ demographic composition from the American Community Survey (ACS) 5-Year estimates for 2017–2021. The variables obtained included total population, median household income, percentage of the population without a college degree, percentage of the population that identified as non-Hispanic Black or African American, percentage of the population that identified as non-Hispanic white, percentage of the population that identified as non-Hispanic American Indian or Alaska Native, percentage of the population that identified as Hispanic (any race), percentage of the population under 18 years of age, and percentage of the population below 150% of the federal poverty line (FPL) – i.e., US$39,750 for a household of four in 2021 [31]. We used the 150% of the FPL threshold to capture not only populations with the lowest income levels, but also those above the poverty line who still experience substantial economic disadvantage, as in previous studies [32,33].

We employed two different measures of rurality. Using 2020 Rural-Urban Commuting Area (RUCA) codes obtained from the U.S. Department of Agriculture Economic Research Service, we classified tracts as urban or rural using a previously established approach [34]. For sensitivity checks, we also calculated tracts’ Index of Relative Rurality (IRR) scores – a continuous and more nuanced indicator of rurality [35] – using data from the U.S. Census.

### Data analysis

Using retailers’ geographic coordinates, we obtained the count of each type of retailer (i.e., alcohol, tobacco, fast food) in each census tract using a geographic overlay. Next, using population estimates provided by ACS, we calculated tracts’ population-based density (i.e., retailers per 1,000 residents) for each type of retailer.

To examine retailer co-location patterns, we first used bivariate Spearman correlation coefficients between population-based density values for each retailer-type pair. Next, we created categorical variables indicating whether a given tract was high in each type of retailer or not – tracts were considered high in a type of retailer if their density values were in the 75^th^ percentile or above for that type of retailer. Tracts were then classified using the following categories: high in none of the retailer types, high in one retailer type only, high in two retailer types only, or high in all three retailer types. We then determined what percentage of North Carolina residents lived in census tracts that were high in none of the retailer types, in only one retailer type, in each grouping of two retailer types, or in all three retailer types. We chose to use categorical variables rather than the number of retailers aggregated across the different lists due to considerable overlap between alcohol and tobacco retailers. Aggregation would have required either double-counting retailers present in more than one list – producing skewed estimates – or collapsing such retailers and erasing their dual purpose.

Lastly, we used bivariate logistic regression models to examine the relationship between tracts’ demographic composition and their probability of being “high in all” retailer types. We employed bivariate rather than multiple regression because the demographic characteristics of interest are highly correlated and conceptually interrelated, and thus our goal was to characterize general associations between retail environments and markers social advantage or disadvantage, rather than to estimate independent effects. Holding other demographic variables constant in multiple regression models would not provide meaningful information, as demographic characteristics do not influence the retail environment independently from each other, but rather through synergistic and mediating mechanisms. After estimating each model, we conducted Moran’s *I* tests on the regression residuals to determine whether spatial autocorrelation should be addressed (S1 Table). For models with potentially problematic residual autocorrelation (*I* < −0.1 or *I* > 0.1), we included a Moran eigenvector on the right-hand side of the logistic regression model. For all spatial tests and calculations, we used queen contiguity-based neighbors. We then used the results from each model to obtain tracts’ predicted probability of being “high in all” retailer types in two scenarios: at the 25^th^ and at the 75^th^ percentiles of the independent variable’s distribution (for continuous variables), or for each category of the independent variable (for the indicator variable).

Primary analyses used the aforementioned population-based retailer density measure given that population size is one of the key factors that attract retailers to a location [36]. However, area-based density may also be a measure of interest in this type of analysis. Therefore, we also calculated the area-based density (i.e., retailers per square kilometer) of each type of retailer and conducted sensitivity checks on our logistic regression models, defining the outcome as whether tracts were classified as “high in all” retailer types according to their area-based retailer density scores.

Spatial analyses were conducted using R version 4.3.1 and statistical analyses were conducted using Stata/BE version 18.

## Results

Table 1 reports characteristics of North Carolina’s census tracts for 2021. Of North Carolina’s 2,672 tracts, 12 had a land area and population of zero in the ACS dataset and zero retailers identified – so we excluded these tracts. Mean population across tracts was 3,936 residents. Retailer counts across tracts ranged from 0 to 22 for alcohol outlets, 0 to 24 for tobacco outlets, and 0 to 44 for fast-food outlets. On average, tracts had 0.99 alcohol retailers, 1.61 tobacco retailers, and 1.53 fast-food retailers per 1,000 residents.

**Table 1:** North Carolina census tracts characteristics (n = 2,660).

		Mean	Median	Range	25^th^ pctl	75^th^ pctl
**Population** (thousands of residents)		3.94	4	0 - 14.78	3	5
**Area** (square kilometers)		47.34	15	0.18 - 1,012.11	5	56
**Retailer count**						
	Alcohol	3.51	3	0 - 22	1	5
	Tobacco	4.06	3	0 - 24	1	6
	Fast Food	3.30	1	0 - 44	0	5
**Population-based retailer density** (i.e., retailers per 1,000 residents, n = 2,649)						
	Alcohol	0.99	0.73	0 - 9.38	0.30	1.39
	Tobacco	1.61	0.86	0 - 1,200	0.38	1.59
	Fast Food	1.53	0.42	0 - 1,600	0.00	1.15
**Area-based density** (i.e., retailers per square kilometer)						
	Alcohol	0.49	0.15	0 - 16.72	0.02	0.63
	Tobacco	0.50	0.15	0 - 12.25	0.03	0.62
	Fast Food	0.58	0.06	0 - 36.76	0.00	0.52
**Percentage below 150% FPL** (n = 2,642)		24.18	22.29	0% − 100%	13.11	32.98
**Median household income** (in thousands, n = 2,629)		64.42	56.74	11.03 - 250	44.76	76.25
**Percentage high school diploma or less** (n = 2,648)		58.11	62.59	0% − 100%	44.55	73.08
**Percentage non-Hispanic white** (n = 2,648)		62.67	68.44	0% − 100%	44.71	83.25
**Percentage non-Hispanic Black or African American** (n = 2,648)		20.95	14.23	0% − 97%	4.56	32.30
**Percentage non-Hispanic American Indian or Alaska Native** (n = 2,648)		1.03	0.00	0% − 94%	0.00	0.33
**Percentage Hispanic** (n = 2,648)		9.39	6.77	0% − 67%	3.03	12.87
**Percentage under 18** (n = 2,648)		21.40	21.55	0% − 49%	17.42	25.78
**Relative rurality (IRR)**		0.53	0.51	0.11 - 0.96	0.37	0.70
		**N**	**%**			
**Binary rurality (RUCA)** (n = 2,652)						
	Not rural	2,411	91%			
	Rural	241	9%			

*Note:* Demographic variables correspond to 5-Year estimates for 2017–2021 from the American Community Survey.

*Note:* Sample size is noted for specific variables when data was not available for the whole sample.

**Fig 1:**
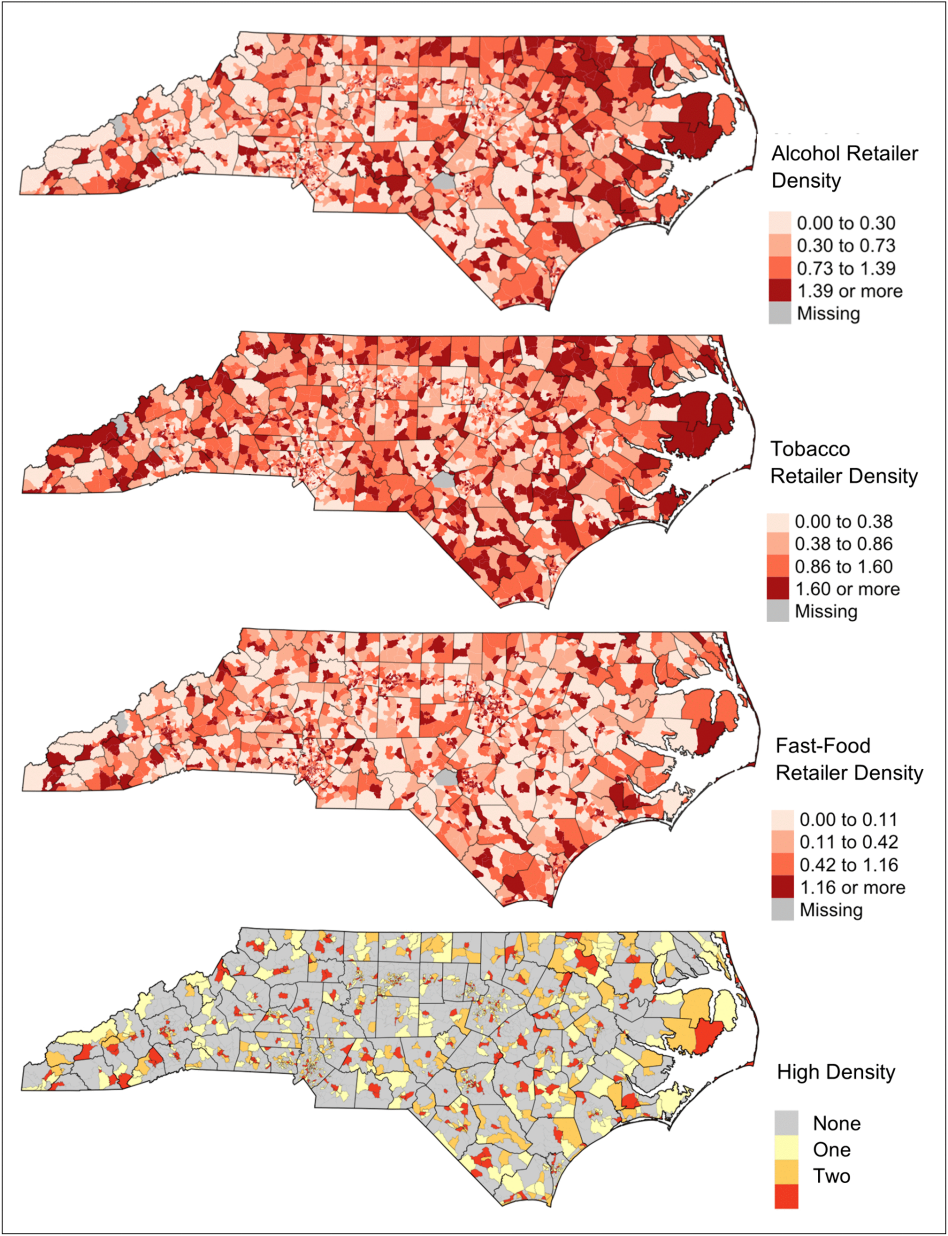
Alcohol, tobacco, and fast-food retailer density* and spatial distribution of tracts with high-density** of one, two, or three retailer types in North Carolina. *Number of retailers per 1,000 residents. **Tracts were considered to have high density for a given type of retailer if its population-based density measure was among the top 25% (75^th^ percentile or above) in North Carolina. Note: Map created using U.S. Census Bureau TIGER/Cartographic Boundary (CB) files (2021), accessed via R (R Foundation for Statistical Computing, Vienna, Austria) using the “tigris” package.

Fig 1 separately displays tracts’ density of alcohol, tobacco, and fast-food retailers. In bivariate correlation analyses, we found alcohol and tobacco density values to be the most strongly correlated (rho = 0.77, p < 0.01), followed by alcohol and fast food (rho = 0.62, p < 0.01) and, lastly, tobacco and fast food (rho = 0.54, p < 0.01). Figure 1 also displays the spatial distribution of tracts classified as high density for one, two, or all three of the retailer types examined.

**Fig 2:**
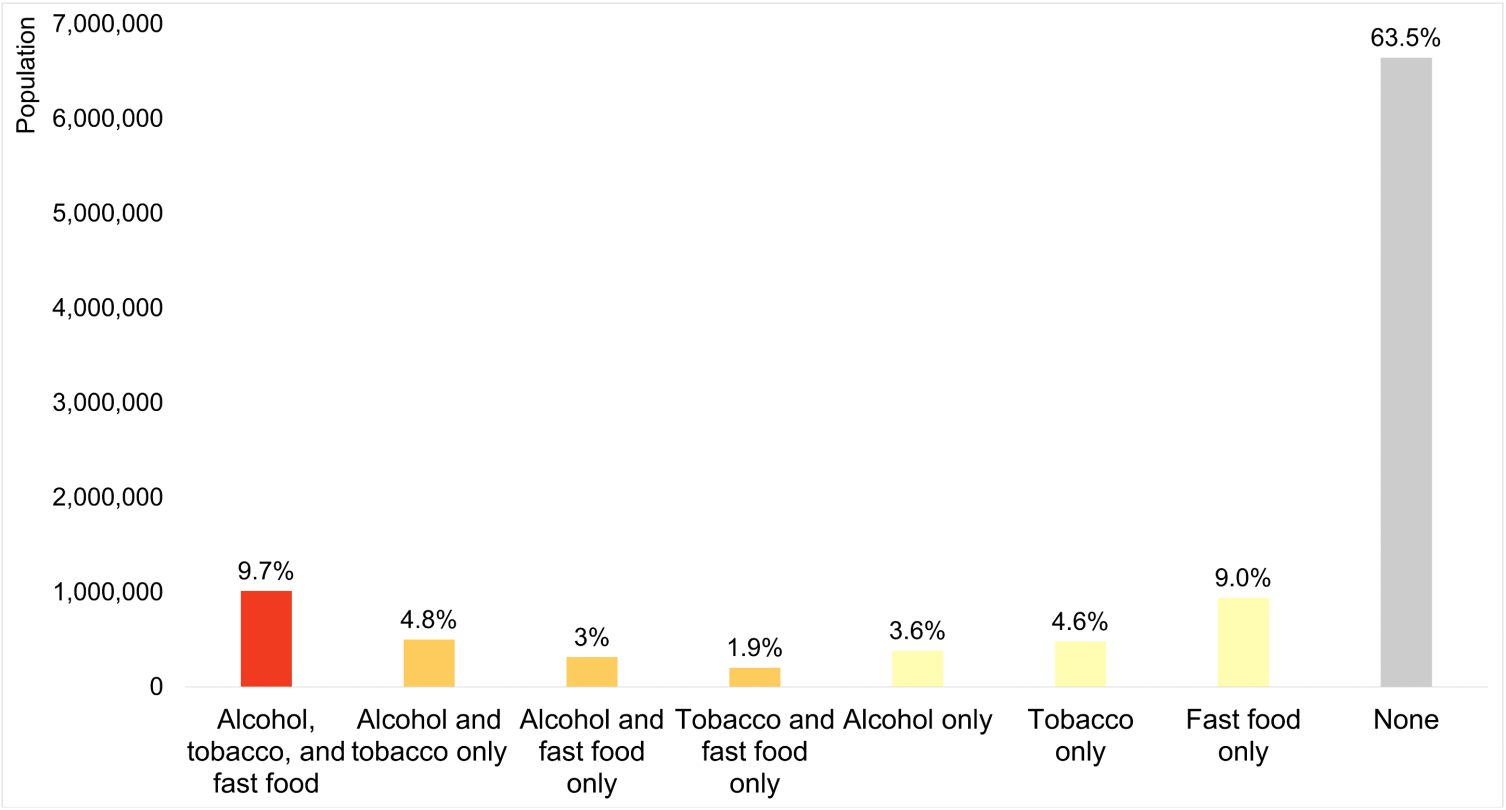
Percentage of North Carolina’s population living in each high-density scenario.

Fig 2 shows the percentage of North Carolina’s population who lived in tracts in each high-density scenario. Around 63.5% of residents lived in tracts not classified as high density for any retailer type, while 36.5% lived in tracts classified as high density for at least one retailer type. Among the latter, the most common scenario was living in tracts classified as “high in all” retailer types, comprising 26.6% of this group and 9.7% of the state’s total population. A nearly as common scenario was living in tracts classified as high density for fast food retailers only, comprising 24.7% of this group and 9% of the state’s total population. Other high-density scenarios were less common, ranging between 4.8% and 1.9% of the state’s population.

Results from bivariate logistic regression models displayed in Table 2 reveal that tracts with a higher percentage of the population living below 150% of the FPL, without a college degree, or that identified as Black or African American had a somewhat higher likelihood of being “high in all” retailer types (all *p* < 0.001, Table 2; Moran’s *I* results reported in S1 Table). Rurality was associated with a higher likelihood of tracts being “high in all” retailer types when measured through the categorical RUCA-based indicator (p = 0.001). However, when rurality was measured on a continuous scale, this association was not significant (p > 0.05). Tracts with a higher median household income (p < 0.001), higher percentage of the population that identified as white (p = 0.005), or higher percentage of the population under 18 years of age (p < 0.001) had a somewhat lower likelihood of being “high in all” retailer types. Alternatively, percentage of the population that identified as Hispanic (any race) or as American Indian or Alaska Native were not significantly associated with tracts’ likelihood of being “high in all” retailer types (both p > 0.05).

**Table 2:** Association between North Carolina census tracts’ demographic characteristics and likelihood having high population-based density for alcohol, tobacco, and fast-food retailers simultaneously.

	OR	SE	95% CI	Prob. at 25th pctl	Prob. at 75th pctl
Percentage below 150% FPL (n = 2,642)	1.03***	0.004	1.03,1.04	7.5%	13.5%
Median household income (n = 2,629)	0.97***	0.003	0.96,0.97	15.6%	6.3%
Percentage high school diploma or less (n = 2,648)	1.02***	0.004	1.01,1.03	8.7%	13.9%
Percentage non-Hispanic Black or African American (n = 2,648)	1.01***	0.003	1.004,1.01	9.8%	12.3%
Percentage non-Hispanic white (n = 2,648)	0.99**	0.002	0.99,0.998	12.5%	9.9%
Percentage American Indian or Alaska Native (n = 2,648)	0.99	0.012	0.97,1.02	11.4%	11.4%
Percentage Hispanic (n = 2,648)	1.01	0.006	0.996,1.02	10.8%	11.6%
Percentage under 18 (n = 2,648)	0.96***	0.009	0.94,0.98	12.7%	9.5%
Relative rurality (IRR) (n = 2,660)	0.61	0.206	0.31,1.18	12.5%	10.7%
				**Prob. for not rural**	**Prob. for rural**
Binary rurality (RUCA)^†^ (n = 2,652)	1.89***	0.34	1.33,2.68	10.8%	18.6%

*Note:* Associations reported based on bivariate models (unadjusted).

†Model including Moran eigenvector on the right-hand to address spatial autocorrelation.

*Statistically significant at the 95% confidence level.

*Statistically significant at the 99% confidence level.

***Statistically significant at the 99.9% confidence level.

Lastly, our sensitivity checks, in which the outcome was defined as whether tracts were classified as “high in all” retailer types according to area-based (rather than population-based) density scores, revealed a few distinct patterns. Specifically, rurality was associated with a *lower* (rather than higher or null) likelihood of tracts being “high in all” retailer types regardless of whether it was measured categorically or continuously. Additionally, having a higher percentage of the population without a college degree was associated with a *lower* (rather than higher) likelihood of being classified as “high in all” retailer types (S2Table).

## Discussion

In this study, we examined the co-location patterns of three types of retailers that substantially expose residents to and increase accessibility of health-harming products – i.e., alcohol, tobacco, and fast-food retailers – in North Carolina, a demographically and geographically diverse US state. Our results revealed that most of the population of this state lived in areas not high in any of the types of retailers examined, likely reflecting predominantly residential areas with limited retail space of any kind. However, among those who lived in an area with high density of any of the retailer types examined, high densities of all three retailer types was the most common scenario. We also found that areas that had a higher percentage of the population living below 150% of the FPL or a higher percentage of the population who identified as Black or African American were somewhat more likely to have high densities of all three retailer types. On the other hand, areas that had a higher median household income, a higher percentage of the population who identified as white, or a higher percentage of the population below 18 years of age were somewhat less likely to have high densities of all three retailer types.

Overall, this study found a substantial degree of co-location among alcohol, tobacco, and fast-food retailers, with high densities of all three retailer types simultaneously being the most common scenario for individuals who lived in an area with high density of any of the retailer types examined. While this pattern may be partially due to retail activity in general clustering in specific areas or hubs due to zoning regulations and market forces, the markedly lower incidence of areas high in only one or only two of the retailer types examined suggests that the co-location of these three types of health-harming retailers may not be random. Notably, the co-location of tobacco and alcohol retailers partially reflects businesses that sell both types of products (e.g., convenience stores, certain pharmacies, certain dollar stores), shown in the strongest correlation between these two retailer types. However, tobacco and alcohol retailer densities were each also positively correlated with density of fast-food retailers, which are mostly separate establishments. Additionally, twice as many people lived in areas high in all three retailer types compared to areas high in alcohol and tobacco only. Future research should explore these patterns in greater depth and in other geographic areas of the US, as well as examine the spatial relationship between alcohol, tobacco, and fast-food retailers and other retailers offering health-promoting products.

Importantly, we also found that certain markers of social disadvantage were linked to a somewhat higher likelihood of areas having high densities of alcohol, tobacco, and fast-food retailers simultaneously. For instance, areas with larger African Americans populations were more likely to have high densities of all three types of retailers, as were areas with larger populations living below 150% of the FPL or with lower median household incomes. Although these associations do not constitute independent effects and their magnitude was not extremely large, when considering that African Americans face the largest burden of several chronic conditions among all racial and ethnic groups in the US [37–40] and that poverty constitutes one of the strongest individual-level predictors of chronic diseases in the country [39–42], our findings underscore the need to better understand the role of retail environments on the health outcomes of socially disadvantaged groups. Specifically, previous research suggests that disadvantaged neighborhood environments may contribute to multimorbidity from chronic conditions above and beyond individual-level determinants [43], and our findings call for a closer examination of how the collective retail landscape may contribute to health-harming neighborhood contexts. Expanding health-related retail landscape analyses on a national scale would be a first key step. Additionally, the limited availability of geographic information (e.g., addresses or census tracts) linked to individual-level health behaviors and outcomes remains an important gap to be filled.

Our findings on the association between areas’ rural status and likelihood of having high densities of all three retailer types were mixed and measure-sensitive. Because rural areas have characteristically small populations and large land areas, our population-based and area-based retail density measures produced conflicting estimates. We contend, however, that exposure to retailers in one’s residential area, which was the focus of this study, is only one construct to consider for rural communities, for whom measures focused on travel and distance to unhealthy retail environments may provide a more accurate assessment of exposure and accessibility. Travel-based analyses were beyond the scope of this study, thus posing a limitation in our ability to draw conclusions about the relationship between rurality and co-location of alcohol, tobacco, and fast-food retailers. Future dedicated investigation on this relationship would be advisable given evidence showing that rural communities face higher rates of chronic conditions compared to urban areas [44].

This study constitutes a key initial step in understanding how health-harming products may cluster in US retail environments. While more studies on this topic are still needed, there are several policy options localities could consider for addressing the clustering of retailers offering health-harming products. For example, zoning regulations and licensing requirements could be linked to measures of retail density that account for multiple categories of health-harming products. Additionally, authorities could offer incentives for the conversion of retail spaces into businesses that provide health-promoting products, such as fruits and vegetables.

Some limitations of this study are worth noting. First, in the absence of a comprehensive list of licensed establishments such as the one available for alcohol retailers, our approach faced some inherent limitations when identifying tobacco and fast-food retailers. Our exclusion of grocery stores as tobacco retailers, while justified by the limited percentage of such stores that sell tobacco, may have somewhat undercounted tobacco retailers. Yet, given the relatively small percentage of tracts high in just fast food and/or alcohol, including grocery stores as tobacco retailers would likely have minimal impact on our overall results. Additionally, because coders were not familiar with all restaurants on our extensive list, for feasibility, we did not require all coders to reach full consensus on whether a business met our fast-food criteria (provided there was no disagreement). In future analyses, involving additional coders could make consensus-coding more feasible, and thus enhance measurement reliability even more. Moreover, because a considerable number of full-service restaurants had three locations or fewer, examining whether these businesses met our fast-food criteria was also not feasible, creating the possibility that independent fast-food restaurants may have been somewhat undercounted. Our results may therefore be conservative estimates, although businesses with such limited presence were not likely to have a large impact on our results. We were also unable to include non-traditional food retailers, such as food trucks, given their changing locations.

This study constituted an ecological analysis, and thus cannot provide conclusions about health behaviors or outcomes; although previous literature suggests that the retail environment influences risk behaviors [7–14]. This study is also unable to infer causality, and thus cannot provide conclusions about what may lead retailers to co-locate; however, such causal inference is not necessarily required for addressing existing issues in retail environments. In addition, the ecological nature of this study’s observation units (Census tracts) makes our statistical findings subject to the Modifiable Areal Unit Problem (MAUP); further research is necessary to evaluate whether grouping the data into alternative areal units, such as Census block groups or counties, would produce similar findings.

Finally, a general limitation of this study is only considering retail exposure in the areas where individuals live, since health behaviors may also be influenced by retail exposures in activity spaces – i.e., spaces where individuals engage in activities such as work, education, or leisure [45]. Recent studies demonstrate that employing human mobility data may be an avenue for reaching a more comprehensive understanding of populations’ exposure to retail environments [46,47], and although this approach is still emerging, it appears to hold potential for future expansion of retail landscape analyses such as the present one.

## Conclusion

This study revealed a substantial degree of co-location of alcohol, tobacco, and fast-food retailers, as well as important disparities in simultaneous exposure to such retailers. This analysis can be adapted to other states and regions, offering important insights into the overlap between retail-related environmental risk factors for chronic diseases that may contribute to health disparities across the United States.

## Supporting information

S1 Table Results from Moran’s I tests using residuals from logistic regression models. (PDF)

S2 Table Association between North Carolina census tracts’ demographic characteristics and likelihood of having high area-based density for alcohol, tobacco, and fast-food retailers simultaneously. *Statistically significant at the 95% confidence level.
(PDF)
