# Supplementary material for: Mapping sources of chronic disease-promoting products in retail environments: An analysis of co-location patterns of alcohol, tobacco, and fast-food retailers
Source: PLoS One. 2026 Apr 20;21(4):e0347097. doi: 10.1371/journal.pone.0347097 (PMC13095102; doi:10.1371/journal.pone.0347097)
Supplement: S1 Table — (PDF) [file pone.0347097.s001.pdf]

Table S1. Results from Moran's I test using residuals from logistic regression models

Model (predictor)	Moran's I	p-value
Percentage below 150% of the poverty line	0.01	0.10
Median household income	0.01	0.14
Percentage without college degree	0.03	0.01
Percentage NH Black or African American	0.04	<0.01
Percentage NH white	0.03	<0.01
Percentage NH American Indian or Alaska Native	0.09	<0.01
Percentage Hispanic	0.03	<0.01
Percentage under 18	0.03	0.01
Relative rurality (IRR)	0.05	<0.01
Binary rurality (RUCA)	0.57	0.00
Binary rurality (RUCA) with spatial adjustment	0.02	0.02
