# Supplementary material for: Mapping sources of chronic disease-promoting products in retail environments: An analysis of co-location patterns of alcohol, tobacco, and fast-food retailers
Source: PLoS One. 2026 Apr 20;21(4):e0347097. doi: 10.1371/journal.pone.0347097 (PMC13095102; doi:10.1371/journal.pone.0347097)
Supplement: S2 Table — *Statistically significant at the 95% confidence level. (PDF) [file pone.0347097.s002.pdf]

**Table S2. Association between North Carolina census tracts' demographic characteristics and likelihood of having high area-based density for alcohol, tobacco, and fast-food retailers simultaneously**

| | OR | SE | 95% CI | Prob. at 25th pctl | Prob. at 75th pctl |
|---|---|---|---|---|---|
| Percentage below 150% of the poverty line | 1.03* | 0.004 | 1.02,1.04 | 11.6% | 19.6% |
| Median household income | 0.99* | 0.002 | 0.98,0.99 | 20.0% | 13.8% |
| Percentage high school diploma or less | 0.99* | 0.003 | 0.98,0.99 | 18.9% | 13.5% |
| Percentage non-Hispanic Black or African American | 1.02* | 0.002 | 1.02,1.03 | 11.5% | 19.1% |
| Percentage non-Hispanic white | 0.98* | 0.002 | 0.97,0.98 | 21.0% | 10.0% |
| Percentage American Indian or Alaska Native | 0.96 | 0.020 | 0.92,1.003 | 16.9% | 16.7% |
| Percentage Hispanic | 1.03* | 0.005 | 1.02,1.04 | 13.5% | 17.7% |
| Percentage under 18 | 0.97* | 0.008 | 0.96,0.99 | 17.9% | 14.8% |
| Relative rurality (IRR) | <0.001* | <0.001 | <0.001,<0.001 | 29.9% | 0.9% |
| | | | | **Prob. for not rural** | **Prob. for rural** |
| Binary rurality (RUCA) | 0.06* | 0.033 | 0.02,0.18 | 18.0% | 1.2% |

*Statistically significant at the 95% confidence level
